# 16QAM Blind Equalization via Maximum Entropy Density Approximation Technique and Nonlinear Lagrange Multipliers

**DOI:** 10.1155/2014/548714

**Published:** 2014-02-27

**Authors:** R. Mauda, M. Pinchas

**Affiliations:** Department of Electrical and Electronic Engineering, Ariel University, 40700 Ariel, Israel

## Abstract

Recently a new blind equalization method was proposed for the 16QAM constellation input inspired by the maximum entropy density approximation technique with improved equalization performance compared to the maximum entropy approach, Godard's algorithm, and others. In addition, an approximated expression for the minimum mean square error (MSE) was obtained. The idea was to find those Lagrange multipliers that bring the approximated MSE to minimum. Since the derivation of the obtained MSE with respect to the Lagrange multipliers leads to a nonlinear equation for the Lagrange multipliers, the part in the MSE expression that caused the nonlinearity in the equation for the Lagrange multipliers was ignored. Thus, the obtained Lagrange multipliers were not those Lagrange multipliers that bring the approximated MSE to minimum. In this paper, we derive a new set of Lagrange multipliers based on the nonlinear expression for the Lagrange multipliers obtained from minimizing the approximated MSE with respect to the Lagrange multipliers. Simulation results indicate that for the high signal to noise ratio (SNR) case, a faster convergence rate is obtained for a channel causing a high initial intersymbol interference (ISI) while the same equalization performance is obtained for an easy channel (initial ISI low).

## 1. Introduction

It is well known that ISI is a limiting factor in many communication environments where it causes an irreducible degradation of the bit error rate, thus imposing an upper limit on the data symbol rate. In order to overcome the ISI problem, an equalizer is implemented in those systems.

Recently [1], a new blind equalization method was proposed for the 16QAM constellation input inspired by the maximum entropy density approximation technique. In this new equalization technique [1], the conditional expectation was approximately derived using Bayes rules where the input and equalized output probability density function (pdf) were approximated using the maximum entropy density approximation technique. In order to find the Lagrange multipliers, a closed-form approximated expression was derived for the MSE where the idea was to bring the MSE to minimum according to the Lagrange multipliers following [2]. Since the obtained expression for the Lagrange multipliers leads to a nonlinear equation for the Lagrange multipliers, the part in the MSE expression that caused the nonlinearity in the Lagrange multipliers' equation was ignored. Thus, a linear closed-form approximated expression was obtained for the Lagrange multipliers from which the proposed Lagrange multipliers [1] were calculated for the 16QAM case.

Up to now, it is not clear whether it is worth finding those Lagrange multipliers that bring the approximated MSE to minimum. Namely, it is not clear if the optimal (from the MSE point of view) Lagrange multipliers derived from the nonlinear equation for the Lagrange multipliers will lead to significant equalization performance improvement or not.

In this paper, we derive the Lagrange multipliers for the 16QAM input case that bring the approximated expression for the MSE to minimum. Namely, we obtain a new set of Lagrange multipliers from the nonlinear equation for the Lagrange multipliers. We show in this paper that the new derived Lagrange multipliers are different in sign and magnitude from the recently obtained set. Simulation results indicate that when we deal with an easy channel, no difference is seen in the equalization performance if we use the Lagrange multipliers derived from the linear or nonlinear equation for the Lagrange multipliers. But, when we deal with a difficult channel (where the initial ISI is considered as very high) and high SNR case, a much faster convergence rate is obtained when using the Lagrange multipliers from the nonlinear equation for the Lagrange multipliers instead of those calculated from the linear part.

The paper is organized as follows. After having described the system under consideration in Section 2, we introduce in Section 3 our new derived Lagrange multipliers for the 16QAM input case. In Section 4, simulation results are presented and the conclusion is given in Section 5.

## 2. System Description

**Figure 1:**
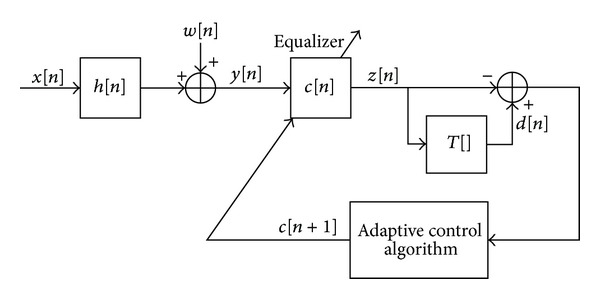
Block diagram of a baseband communication system.

The system under consideration is the same system used in [1], illustrated in Figure 1, where we make the following assumptions as used in [1].

(1) The input sequence *x*[*n*] belongs to the 16QAM constellation where *x*_1_[*n*] and *x*_2_[*n*] are the real and imaginary parts of *x*[*n*], respectively. (2) The unknown channel *h*[*n*] is a possibly nonminimum phase linear time-invariant filter in which the transfer function has no “deep zeros”; namely, the zeros lie sufficiently far from the unit circle. (3) The equalizer *c*[*n*] is a tap-delay line. (4) The noise *w*[*n*] is an additive Gaussian white noise. (5) The function *T*[·] is a memoryless nonlinear function that satisfies the analyticity condition: *T*[*z*_1_[*n*] + *jz*_2_[*n*]] = *T*[*z*_1_[*n*]] + *jT*[*z*_2_[*n*]], where *z*_1_[*n*], *z*_2_[*n*] are the real and imaginary parts of the equalized output, respectively.

The sequence *x*[*n*] is transmitted through the channel *h*[*n*] and is corrupted with noise *w*[*n*]. Convolving *c*[*n*] with the received sequence, we obtain

(1) $$\begin{array}{c} z\left[n\right]=x\left[n-D\right]e^{j\theta }+p\left[n\right]+\tilde{w}\left[n\right], \end{array}$$

where *D* is a constant delay, *θ* is a constant phase shift, and *p*[*n*] is the convolutional noise, namely, the residual intersymbol interference (ISI) arising from the difference between the ideal and the guess value for *c*[*n*] and $\tilde{w}\left[n\right]=w\left[n\right]*c\left[n\right]$, where “∗” denotes the convolution operation. In this paper (as it was done in [1]), we assume that *D* = 0 and *θ* = 0, since *D* does not affect the reconstruction of the original input sequence *x*[*n*] and *θ* can be removed by a decision device [3]. The ISI is often used as a measure of performance in equalizers' applications, defined by

(2) $$\begin{array}{c} \text{ISI}=\frac{\sum_{\tilde{m}}{\left|{\tilde{s}}_{\tilde{m}}\right|}^{2}-{\left|\tilde{s}\right|}_{\max}^{2}}{{\left|\tilde{s}\right|}_{\max}^{2}}, \end{array}$$

where $|\tilde{s}{|}_{\max}$ is the component of $\tilde{s}$, given in (3), having the maximal absolute value:

(3) $$\begin{array}{c} \tilde{s}\left[n\right]=c\left[n\right]*h\left[n\right]=\delta \left[n\right]+\xi \left[n\right], \end{array}$$

where *δ* is the Kronecker delta function and *ξ*[*n*] stands for the difference (error) between the ideal and the guess value for *c*[*n*]. Next, we define some estimator of *x*[*n*], *d*[*n*] which is produced by the function *T*[*z*[*n*]]. Thus, the error signal is $\tilde{e}\left[n\right]=T\left[z\left[n\right]\right]-z\left[n\right]$. This error is fed into the adaptive mechanism which updates the equalizer's taps. The conditional expectation (*E*[*x*[*n*]/*z*[*n*]], where *E*[·] stands for the expectation operation) is touted as a good estimate of *T*[*z*[*n*]] [3].

According to [1], the equalizer's taps are updated according to

(4) cl[n+1]=cl[n]−μWy∗[n−l]withW=E[x1z1][(z1[n]E[x1/z1])〈(z1)2〉n]  +jE[x2z2][(z2[n]E[x2/z2])〈(z2)2〉n]−z[n],

where *μ* is a positive stepsize parameter, (·)* stands for the conjugate operation on (·), *l* stands for the *l*th tap of the equalizer, *x*_1_ = *x*_1_[*n*], *x*_2_ = *x*_2_[*n*], *z*_1_ = *z*_1_[*n*], *z*_2_ = *z*_2_[*n*], and

(5) E[xs[n]zs[n]]≅zs[n]+g1′′(zs)2g(zs)(σzs2−σxs2) +g1′′′′(zs)    8g(zs)(σzs2−σxs2)2,

where  

(6) s=1,2;g(zs)=exp⁡(∑k=2Kλkszs[n]k‍),g1′′(zs)={d2dxs2[xsexp⁡⁡(∑k=2Kλksxsk‍)]}xs=zs[n],g1′′′′(zs)={d4dxs4[xsexp⁡⁡(∑k=2Kλksxsk‍)]}xs=zs[n]

and *λ*_*k*_^1^, *λ*_*k*_^2^ are the Lagrange multipliers related to *x*_1_[*n*] and *x*_2_[*n*], respectively, and *σ*_*x*_1__^2^, *σ*_*x*_2__^2^ are the variances of the real and imaginary parts of the source signal, respectively. The variances of the real and imaginary parts of the equalized output signal are defined as *σ*_*z*_1__^2^ and *σ*_*z*_2__^2^, respectively, and may be estimated by [1]

(7) $$\begin{array}{c} {\langle z_{s}^{2}\rangle }_{n}=\left(1-\beta \right){\langle z_{s}^{2}\rangle }_{n-1}+\beta \cdot {\left(z_{s}\right)}_{n}^{2}, \end{array}$$

where 〈·〉 stands for the estimated expectation, 〈*z*_*s*_^2^〉_0_ > 0, and *β* is a positive stepsize parameter.

## 3. New Lagrange Multipliers

In this section, we present the Lagrange multipliers for the 16QAM input case that brings the approximated MSE derived in [1] to minimum. Since we are dealing with the 16QAM case, the MSE (MSE = *E*[(*E*[*x*[*n*]/*z*[*n*]] − *x*[*n*])(*E*[*x*[*n*]/*z*[*n*]] − *x*[*n*])*]) is twice the MSE obtained for the real valued case.

According to [1], the approximated MSE for the real valued case is given by

(8) MSE≅E[((z1[n]−x1[n])+g1′′(z1)2g(z1)σp2+g1′′′′(z1)8g(z1)σp4)2]  ≅σp2+σp4∑k=2K  (k2+k)λk(k−1)mk−2‍   +σp4∑l=2L∑ k=2Klkλlλk(k+l−1)mk+l−2‍  ‍   +σp44E[(g1′′(x1)g(x1))2],

where

(9) σp44E[(g1′′(x1)g(x1))2] =σp44∑l=2 K∑k=2 K∑q=2 K∑t=2Kkltqλlλkλqλtmk+l+t+q−2‍  ‍    +σp42∑l=2 K∑k=2 K∑r=2 Kkl(r2+r)λlλkλrmk+l+r−2‍‍    +σp44∑k=2 K∑l=2K(k2l2+2k2l+kl)‍‍  λkλlmk+l−2,

where *m*_*k*_ = *E*[*x*_1_^*k*^[*n*]],  *σ*_*p*_^2^ = *E*[*p*_1_^2^[*n*]] = *σ*_*z*_1__^2^ − *σ*_*x*_1__^2^ (where *p*_1_[*n*] is the real part of *p*[*n*]), and *λ*_*k*_ (*k* = 1,2, 3 …, *K*) are the Lagrange multipliers. Next, we wish to find those Lagrange multipliers that bring the MSE (8) to minimum. Namely, we are looking for those Lagrange multipliers that comply with the following equation: ∂MSE/∂*λ*_*k*_ = 0. By carrying out ∂MSE/∂*λ*_*k*_ = 0 (*k* = 1,2, 3,…, *K*) and taking into account that *E*[*x*[*n*]] = 0 we obtain the following expression:

(10) (k2+k)(k−1)mk−2+2k2(2k−1)λkm2k−2 +2∑l=2, l≠kKlkλl(k+l−1)mk+l−2+∂A1∂λk +∂A2∂λk+∂A3∂λk=0 for  k=2,4,6,…,K,

where

(11) A1=14∑l=2K∑k=2K∑q=2K∑t=2Kkltqλlλkλqλtmk+l+t+q−2‍  ‍  ‍‍A2=12∑l=2 K∑k=2 K∑r=2Kkl(r2+r)λlλkλrmk+l+r−2‍  ‍  ‍A3=14∑k=2 K∑l=2K‍  (k2l2+2k2l+kl)λkλlmk+l−2.‍  

For the 16QAM constellation input case we use two Lagrange multipliers *λ*_2_, *λ*_4_ as was done in [2], thus having *k* = 2,4 where *K* = 4. By using (10) we obtain the following equations for *λ*_2_ and *λ*_4_:

(12) $$\begin{array}{c} 6+24\lambda_{2}m_{2}+80\lambda_{4}m_{4}+\frac{\partial A_{1}}{\partial \lambda_{2}}+\frac{\partial A_{2}}{\partial \lambda_{2}}+\frac{\partial A_{3}}{\partial \lambda_{2}}=0,\\ 60m_{2}+224\lambda_{4}m_{6}+80\lambda_{2}m_{4}+\frac{\partial A_{1}}{\partial \lambda_{4}}+\frac{\partial A_{2}}{\partial \lambda_{4}}+\frac{\partial A_{3}}{\partial \lambda_{4}}=0. \end{array}$$

Please note that the following expressions ∂*A*_1_/∂*λ*_2_ + ∂*A*_2_/∂*λ*_2_ + ∂*A*_3_/∂*λ*_2_ and ∂*A*_1_/∂*λ*_4_ + ∂*A*_2_/∂*λ*_4_ + ∂*A*_3_/∂*λ*_4_ were set to zero when the Lagrange multipliers were derived in [1]. According to [1], *λ*_2_ = −0.52095; *λ*_4_ = 1.7230 × 10^−2^. But by solving the nonlinear equation (12) with MATLAB software we obtain

(13) $$\begin{array}{c} \lambda_{2}=0.7904995,\;\;\lambda_{4}=-0.033887. \end{array}$$

As we may see, the new values for *λ*_2_ and *λ*_4_ are very different (opposite in sign and different in magnitude) from those obtained in [1]. Thus, it is not clear whether the new values for *λ*_2_ and *λ*_4_ will lead to improved equalization performance compared with the previous obtained set or not. In the next section, we will try to answer on that issue.

## 4. Simulation

In this section, the equalization performance with the new values for *λ*_2_ and *λ*_4_ was investigated by simulation. For that purpose we used Godard's [4] algorithm, the maximum entropy algorithm [2], and the previously derived values [1] for *λ*_2_ and *λ*_4_ for comparison. In the following, we denote “MaxEnt,” “MaxEnt_New_,” and “MaxEnt_nonlinear_” as the algorithm described in [2], (4) with *λ*_2_ = −0.52095; *λ*_4_ = 1.7230 × 10^−2^, and (4) with (13), respectively. The step-size parameters for the “MaxEnt_nonlinear_” algorithm are defined as *μ*_nonlinear_ and *β*_nonlinear_. The step-size parameters for the “MaxEnt” algorithm are defined as *μ*_Ent_ and *β*_Ent_. The step-size parameters for the “MaxEnt_New_” algorithm are defined as *μ*_EntNew_ and *β*_EntNew_ and the step-size parameter for Godard's algorithm is defined as *μ*_*G*_. For the “MaxEnt,” “MaxEnt_New_,” and “MaxEnt_nonlinear_” algorithm we used *E*[*z*_*s*_^2^] = *E*[*x*_*s*_^2^] for initialization. Two different channels were considered.

*Channel 1 (initial *ISI = 0.44*).* Taken according to [5], *h*_*n*_ = {0  for  *n* < 0; −0.4  for  *n* = 0; 0.84 · 0.4^*n*−1^ for *n* > 0}.

**Figure 2:**
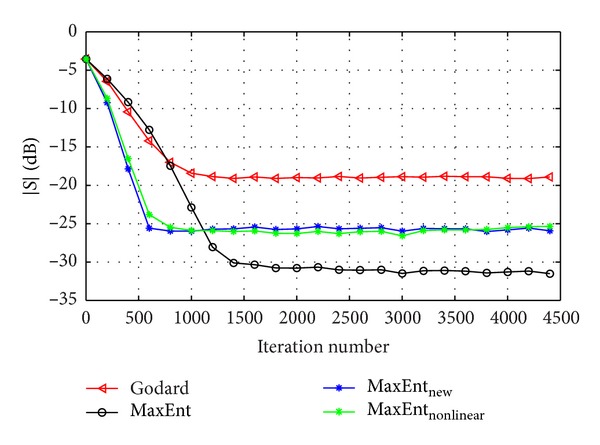
Performance comparison between equalization algorithms for a 16QAM source input going through channel 1. The averaged results were obtained in 100 Monte Carlo trials for SNR = 30 dB. *μ*_nonlinear_ = 6∗10^−4^, *β*_nonlinear_ = 2∗10^−5^, *μ*_Ent_ = 3∗10^−4^, *β*_Ent_ = 2∗10^−5^, *μ*_EntNew_ = 1∗10^−3^, *β*_EntNew_ = 1∗10^−4^, and *μ*_*G*_ = 6∗10^−5^.

**Figure 3:**
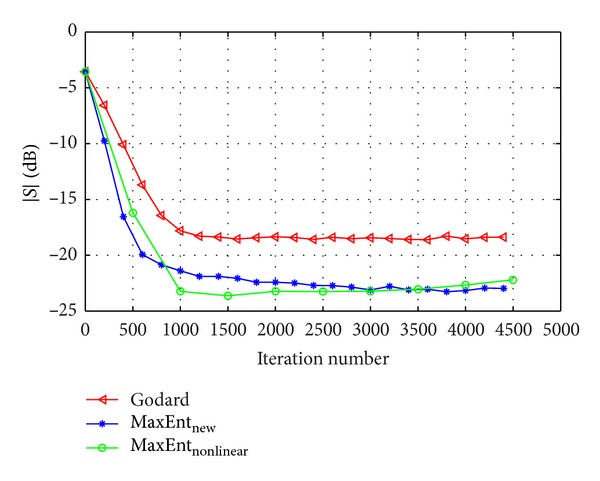
Performance comparison between equalization algorithms for a 16QAM source input going through channel1. The averaged results were obtained in 100 Monte Carlo trials for SNR = 20 dB. *μ*_nonlinear_ = 6∗10^−4^, *β*_nonlinear_ = 2∗10^−5^, *μ*_EntNew_ = 1∗10^−3^, *β*_EntNew_ = 1∗10^−4^, and *μ*_*G*_ = 6∗10^−5^.

**Figure 4:**
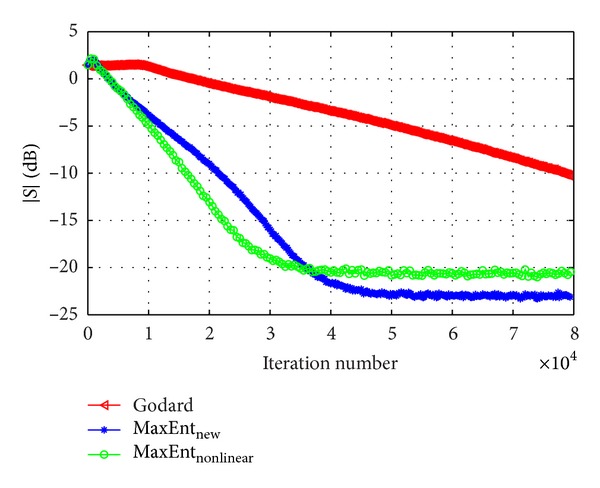
Performance comparison between equalization algorithms for a 16QAM source input going through channel 2. The averaged results were obtained in 50 Monte Carlo trials for SNR = 30 dB. *μ*_nonlinear_ = 4∗10^−5^, *β*_nonlinear_ = 5∗10^−8^, *μ*_EntNew_ = 0.00035, *β*_EntNew_ = 5∗10^−6^, and *μ*_*G*_ = 1∗10^−5^.

*Channel 2 (initial *ISI = 1.402*).* Taken according to [6], *h*_*n*_ = (0.2258,0.5161,0.6452,0.5161). For Channel 1 and Channel 2, we used an equalizer with 13 and 21 taps, respectively. The equalizers were initialized by setting the center tap equal to one and all others to zero. The step-size parameters *μ*_nonlinear_, *β*_nonlinear_, *μ*_ENT_, *β*_ENT_, *μ*_EntNew_, *β*_EntNew_, and *μ*_*G*_ were chosen for fast convergence with low steady state ISI. Figures 2 and 3 show the equalization performance with the new derived Lagrange multipliers (13), namely, the ISI as a function of iteration number for the 16QAM constellation input sent via channel1 for SNR = 30 [dB] and SNR = 20 [dB], respectively, compared with the equalization performance obtained from the maximum entropy [2], Godard's [4], and MaxEnt_New_ algorithm. According to simulation results (Figures 2 and 3), our new proposed algorithm (4) with the Lagrange multipliers defined in (13) has a much faster convergence time, compared with the maximum entropy [2] and Godard's [4] algorithm, but has approximately the same equalization performance as the MaxEnt_New_ algorithm. Namely, the new and previously obtained Lagrange multipliers lead approximately to the same equalization performance. Thus, there is no advantage by using the new Lagrange multipliers over the previously obtained set.  Figure 4 shows the equalization performance with the new derived Lagrange multipliers (13), namely, the ISI as a function of iteration number for the 16QAM constellation input sent via channel 2, compared with the equalization performance obtained from the MaxEnt_New_ and Godard's [4] algorithm. According to simulation results (Figure 4), our new proposed algorithm (4) with the Lagrange multipliers defined in (13) has better equalization performance, namely, a much faster convergence time, compared with the MaxEnt_New_ and Godard's [4] algorithm. As a matter of fact, the convergence speed of the equalizer with the new proposed Lagrange multipliers (13) is faster with approximately 5000 symbols (the eye-diagram is already open at ISI≅−16 [dB]) and with over 30000 symbols compared with the equalizer with the previously obtained Lagrange multipliers and Godard's [4] algorithm, respectively. Thus, the new proposed Lagrange multipliers (13) are more attractive than the previously derived set.

## 5. Conclusion

In this paper, we derived new Lagrange multipliers for the 16QAM input case that bring the approximated MSE to minimum. Simulation results have shown that when we deal with an easy channel, no difference is seen in the equalization performance if we use the new or previously obtained Lagrange multipliers. But, when we deal with a difficult channel (where the initial ISI is considered as very high) and high SNR case, a much faster convergence rate is obtained when using our new proposed Lagrange multipliers over the previously derived set.
